# Mathematical recapitulation of the end stages of human ovarian aging

**DOI:** 10.1126/sciadv.adj4490

**Published:** 2024-01-12

**Authors:** Sean D. Lawley, Mary D. Sammel, Nanette Santoro, Joshua Johnson

**Affiliations:** ^1^ Department of Mathematics, University of Utah, 155 S 1400 E, JWB 233, Salt Lake City, UT 84112, USA.; ^2^ Department of Biostatistics and Informatics, Colorado School of Public Health, Aurora, CO 80045, USA.; ^3^ Department of Obstetrics and Gynecology, University of Colorado School of Medicine (AMC) Building RC2, Room P15 3103, Aurora, CO 80045, USA.

## Abstract

Ovarian aging in women can be described as highly unpredictable within individuals but predictable across large populations. We showed previously that modeling an individual woman’s ovarian reserve of primordial follicles using mathematical random walks replicates the natural pattern of growing follicles exiting the reserve. Compiling many simulations yields the observed population distribution of the age at natural menopause (ANM). Here, we have probed how stochastic control of primordial follicle loss might relate to the distribution of the preceding menopausal transition (MT), when women begin to experience menstrual cycle irregularity. We show that identical random walk model conditions produce both the reported MT distribution and the ANM distribution when thresholds are set for growing follicle availability. The MT and ANM are shown to correspond to gaps when primordial follicles fail to grow for 7 and 12 days, respectively. Modeling growing follicle supply is shown to precisely recapitulate epidemiological data and provides quantitative criteria for the MT and ANM in humans.

## Teaser

Modeling the loss of ovarian follicle reserve produces the population distributions of menopause and the menopausal transition.

## INTRODUCTION

Menopause is defined as having occurred when 12 months have passed without a menstrual period and is now understood to result as a consequence of changes that take place within the hypothalamic-pituitary-ovarian axis (*1*). Individuals can achieve the state at any time, with an upper limit around age 62. The overall distribution centered around a median age of 51, however, is highly consistent between studies and does not vary greatly between distinct ethnic groups (*2*). Before menopause, menstrual cycles become irregular and a constellation of physiologic changes occur that together are referred to as the menopausal transition (MT) (*3*–*5*). The existing criteria used to define menopause and the MT reflect altered signaling within the hypothalamic-pituitary-ovarian axis and correspond to declining numbers of ovarian follicles, but definitive upstream causative events that trigger their onset remain elusive.

**Fig. 1.**
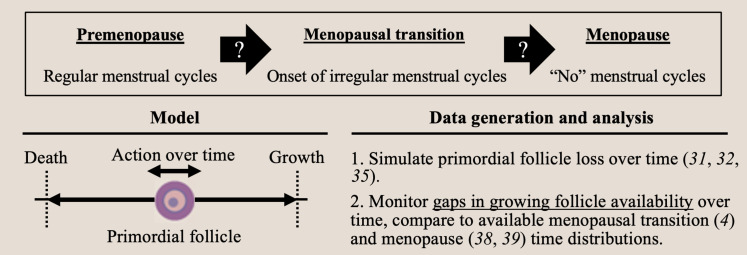
Concept of the study. This work evaluates whether stochastic loss of primordial ovarian follicles determines the timing of onset (question marks, black arrows) of the MT and ANM. The decision for primordial follicles to stay dormant or to begin to grow is modeled using mathematical random walks relative to growth and death thresholds (bottom left). In this study (bottom right), the numbers of follicles matching the reported distribution for girls at birth underwent random walks and the gaps in follicle growth activation (e.g., the supply of growing follicles) were monitored. Thresholds for gap length were then considered relative to the reported MT and ANM population distributions.

At birth, the primordial ovarian follicle (PF) reserve “starting supply” begins within the range of 10^5^ to 10^6^ PFs. The reserve then decreases over the next several decades as individual PFs begin to grow and progress through several stages of development (*6*, *7*). Evidence supporting a positive relationship between the starting supply of PFs at birth in girls and the overall duration of adult ovarian function is available (*8*, *9*). In addition, mechanisms have been identified that determine whether individual follicles begin to grow and how follicle death occurs (*10*–*15*), but little information is available that shows how the overall patterns of follicle decline over time, and the timing of the onset of the MT and menopause are ultimately controlled (Fig. 1).

It is clear, however, that the vast majority of follicles (≈99.9%) die before ovulation (*16*–*21*), while only about one growing follicle survives to ovulate per month (termed follicle “selection”) throughout the reproductive years until the MT [onset of irregular cycles; (*4*, *22*)] and then menopause (cessation of menses for 12 months) are reached. Within individual women, the MT is characterized by menstrual cycle irregularity, which can manifest as shorter or longer intermenstrual intervals that can include months of amenorrhea (*23*). The MT entails “unpredictable menstrual cycle endocrine patterns” (*24*), and, late in the MT, about half of cycles are anovulatory (*24*). Between women, the MT differs in age at onset (ranging from ages 42 and 54 for most women) and duration (2 to 10 years for most women) (*4*). The timing of onset of the age at natural menopause (ANM) is associated with a dwindling ovarian reserve of PFs and occurs when women have approximately 10^3^ PFs remaining (*25*).

Ovarian biologists have long noted that the loss of PFs over time resembles radioactive decay and could even involve stochastic mechanisms (*26*, *27*). In accordance with this, our recent wet laboratory and bioinformatics results have identified possible physiological sources of stochasticity within mammalian ovaries (*15*, *28*). One proposed source of randomness is the cellular response to damage by the integrated stress response pathway (*29*, *30*). Specifically, we have proposed that integrated stress response checkpoint resolution (e.g., when ongoing physiological stress and DNA damage are resolved) in individual PFs allows entrance into the cell cycle and thus triggers PF growth activation (PFGA). Modeling PF response to integrated stress response pathway-activating stressors by a stochastic process [as supported by a variety of wet lab measurements; (*15*, *28*)] results in a stochastic PFGA time for each PF. Random walk modeling of PFGA in this fashion results in the accurate recapitulation of patterns of PF loss seen in individual women and also an accurate recapitulation of the distribution of the ANM (*31*, *32*) in the population when we incorporate the well-known population variability in PF starting supply at birth (*33*, *34*). Instead of complex signaling mechanisms that select which follicles live and which die, stochastic control of follicle growth and death allows relatively simple rules to give rise to the known patterns of follicle loss and survival emergently (*15*, *28*, *31*, *32*, *35*). The involvement of stochastic mechanisms leads to a plausible explanation for what has been viewed as an “oversupply” of primordial follicles at birth and the robust function of ovaries for decades after menarche (*32*, *35*).

In this work, we study how numbers of remaining PFs might relate causally to the timing of the landmark events of ovarian aging, the onset of the MT, and the 12 months without menses that heralds menopause. We first tested whether random walk modeling of PF behavior could also give rise to any pattern(s) reminiscent of the MT onset time distribution. Following that, we tested whether thresholds of growing follicle availability (due to PFGA) is related to the timing of the onset of the MT and/or menopause. Results here further support the involvement of biological stochasticity in ovarian aging and inform our mechanistic understanding of the causal origins of the timing of the MT and the ANM in individuals and populations of women.

## RESULTS

### Stochastic gaps in PFGA mark a declining ovarian reserve

Assuming a stochastic, exponential decay of PFs in the reserve [as in (*16*, *31*, *36*, *37*)]; see Materials and Methods for mathematical details) implies that the time between PFGA events is roughly exponentially distributed with rate λ*R*, where *R* is the current size of the reserve and λ is the per follicle PFGA rate. This implies that, as the reserve size *R* decreases over postpartum life, the time between PFGA events increases both in mean and SD. On the basis of our recent random walk model of ovarian aging (*31*), we estimate that λ ≈ 0.17/year. Therefore, when the reserve size is at least tens of thousands of PFs, a woman will have many PFGAs every day, and there is virtually no chance that she will go more than a day without PFs beginning to grow. On the other hand, as the reserve size depletes to only several thousand, a woman may sometimes go several days without a newly growing follicle.

**Fig. 2.**
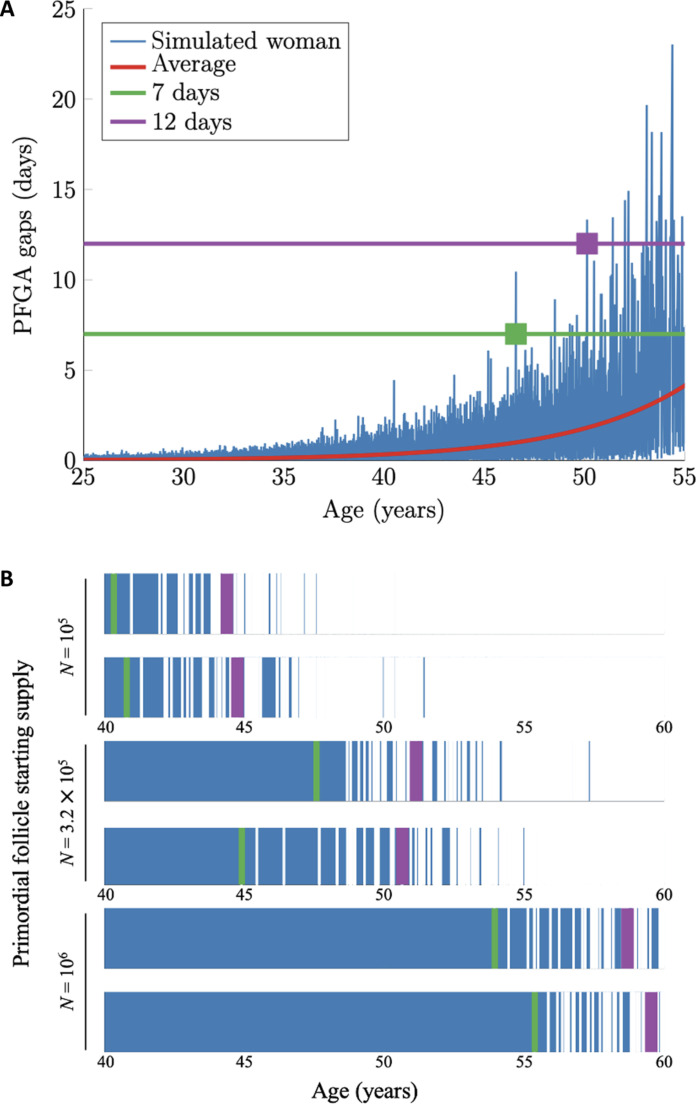
Stochastic PFGA results in gaps where zero follicles begin to grow. An example trace of gaps in growing follicle availability between ages 25 and 55 for a single simulated individual is shown in (**A**). The average number of days where zero PFs begin to grow is shown by the red line, and gaps experienced by an individual are shown in blue. *Y*-axis thresholds are shown for 7-day (green) and 12-day (purple) gaps given detected relationships between these gap lengths and onset of the menopausal transition and menopause, respectively. (**B**) Example barcode plots for six unique simulations. Two examples are provided each, where ovarian reserve at birth is the lowest first percentile (10^5^), the population median (3.2 × 10^5^), or the highest first percentile (10^6^) (*y* axis). Days that PFGA occurs are blue, and gaps where no PFs begin to grow are white. Green and purple bands indicate the first time gaps of 7 and 12 days occur in each simulation.

In Fig. 2A, we have modeled a single simulated woman with median ovarian reserve and plot the time between PFGA events between the ages of 25 and 55 years. We refer to these as PFGA “gaps” or “gaps in the growing follicle supply.” PFGA gaps are all much less than 1 day until she reaches her thirties, which is when her reserve starts to dip below a few tens of thousands. As she reaches her late thirties and early forties and the reserve decreases to several thousand, PFGA gaps of more than 1 day become frequent. Gaps of around a week then become common in her late forties when her reserve is just over 1000. Last, gaps in the growing follicle supply of 2 weeks or more characterize her early fifties when the reserve dips below 1000 and approaches several hundreds. Furthermore, Fig. 2A shows that stochastic fluctuations in PFGA gaps often differ substantially from their mean (mean PFGA gap is plotted in red in Fig. 2A). Although the mean gap does not rise above 1 day until age 46 (corresponding to a reserve size of about 2000 PFs), gaps of several days are already common by this age.

To further illustrate the impact of stochasticity on gaps in the growing follicle supply, in Fig. 2B, we plot PFGA patterns as “barcode” plots for unique women with different starting supplies of PFs at birth (PF starting supply is denoted by *N*). Each plot displays ages 40 to 60 years, where blue indicates time ranges when PFs begin to grow and white indicates gaps when no PFs begin to grow for 7 or more days. Plots corresponding to two unique simulated women with PF starting supplies at the lowest first percentile (top two plots, PF starting supply *N* = 10^5^), the population median (middle two plots, *N* = 3.2 × 10^5^), or the top first percentile (bottom two plots, *N* = 10^6^) are provided where the PFGA rate λ is identical. Stochastic PFGA results in differing times of gap onset between simulated women, and there is a clear positive relationship between the ages that gaps in growing follicle supply occur and the starting supply of PF reserve size at birth (*31*, *33*) in girls.

### MT onset timing coincides with the first PFGA gap of 7 days

**Fig. 3.**
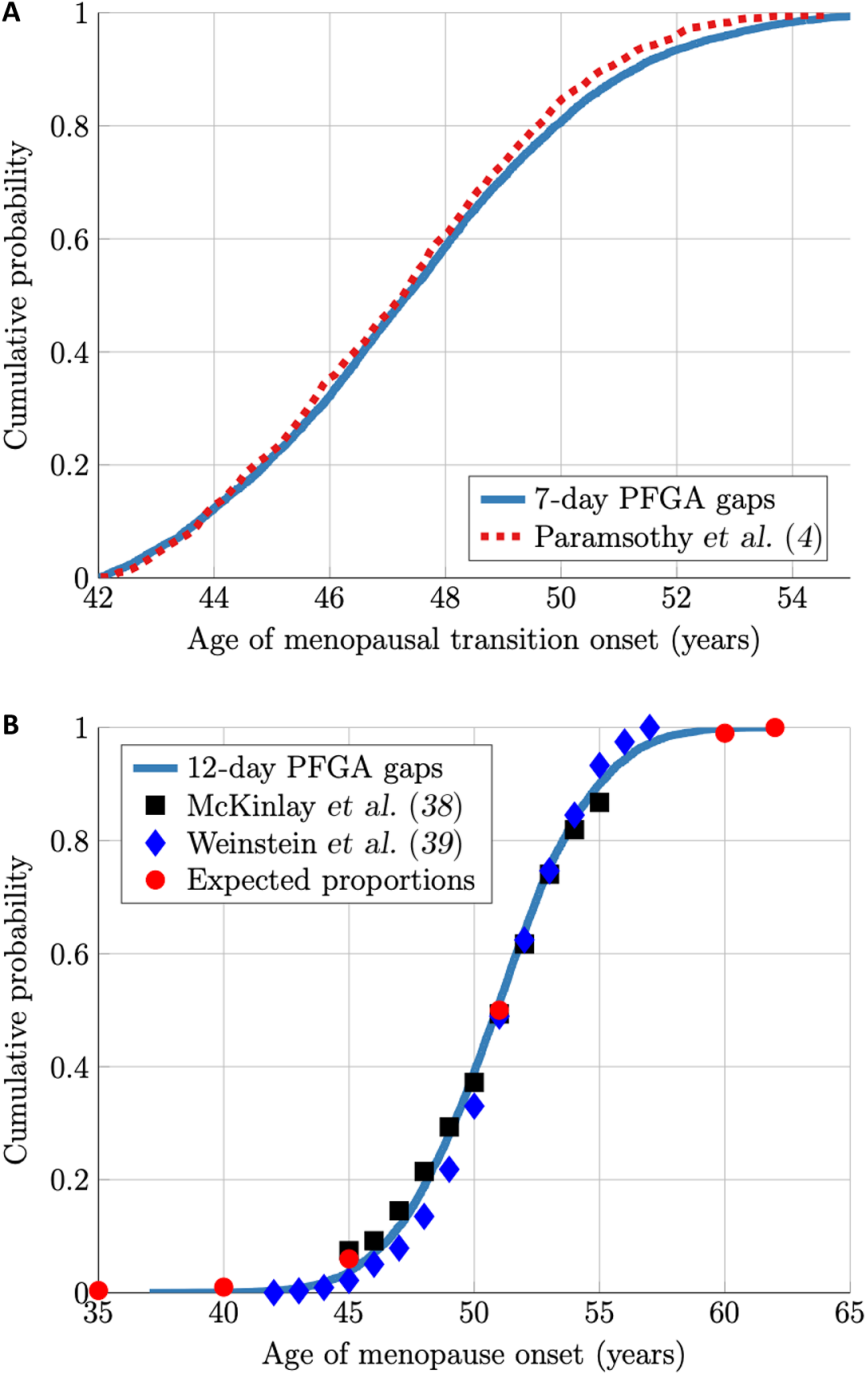
Identical stochastic model conditions recapitulate the reported menopausal transition and menopause distributions. Growing follicle supply gap length data from simulated individuals was compiled and is reported as cumulative distribution plots. (**A**) A comparison between simulation output where the first gap length of 7 days was compiled (blue solid line) and menopausal transition onset data reported by (*4*) (red dashed line). (**B**) Comparison of compiled simulation output for gaps of 12 days (blue solid line) and menopause onset data from (*38*, *39*) (blue and black symbols), as well as “expected proportions” from the literature (red dots). Note that the cumulative probability distribution for the stochastic simulation where the 12-day gap length threshold was used is indistinguishable from simulations using a threshold of 1000 remaining primordial follicles as in (*31*).

Given that an interrupted supply of growing ovarian follicles would interrupt the endocrine feedback that dictates menstrual cycles, we hypothesized that sufficiently large PFGA gaps would correspond to and thus “trigger” the MT. Concretely, consider the first time *T*_MT_ that there is a gap larger than a critical threshold of *t*_1_ days. Interrogation of gap sizes in simulation data led to the identification of a gap length threshold of 7 days that matched available MT onset distribution data (below, Fig. 3A) where “a persistent difference of at least 7 days in the length of consecutive menstrual cycles” (*4*, *22*) occurred. The green horizontal line in Fig. 2A marks this “MT threshold,” and the green square indicates the first time this simulated individual experiences a gap length that exceeds 7 days. We found that compiled simulations using a gap length of 12 days (purple line) matched the ANM distribution (below, Fig. 3B), and the purple square indicates the time when this individual experienced that gap in growing follicle supply. Green and purple bands in the barcode plots in Fig. 2B indicate the times when those same gap lengths are reached by the six simulated individuals.

In Fig. 3A, we compare (i) the distribution of *T*_MT_ computed from stochastic simulations of the (random walk) model (*31*) across a population of 10^4^ simulated women with (ii) the empirical population distribution of the MT onset from (*4*) (a study of 1415 women whose MT onset and length were monitored). In this plot, the only “fitted” parameter for simulation-derived data is the threshold *t*_1_ = 7 days, as the random walk model was based on PF count data (*31*). We note here that the data from (*4*) excluded the small fraction of women who started their MT before age 42, and, so in Fig. 3A, we similarly excluded simulated women who reached the MT before age 42. With this caveat in mind, we can say that *t*_1_ = 7 days is the threshold number of days that can pass with zero PFGA (e.g., where a “gap” in growing follicle supply occurs for 7 days) that corresponds to the onset of the MT.

On the basis of these findings, we propose that stochastic gaps in the supply of growing follicles disrupt the endocrine signaling required for regular cycles and thusly triggers the MT. MT onset can therefore be defined as occurring when the growing follicle supply is not consistent enough to (e.g., stochastically crosses the threshold that can) support regular menstrual cycles.

### Menopause coincides with the first PFGA gap of 12 days

Treatment of the MT in this way also allowed us to revisit the question of the timing of the ANM. While setting a threshold of 10^3^ remaining PFs resulted in a satisfying recapitulation of the ANM distribution (*31*), it is unlikely that the round number of 10^3^ is physiologically meaningful but is instead an approximation across women. For this reason, we attempted to determine a threshold for the ANM that was also based on the gap length between PFGA events. Like the MT, there was a threshold for the number of days that pass with zero PFGA (zero growing follicle supply) that strongly corresponds to the ANM distribution, and this was found to be 12 days. In Fig. 3B, we compare the distribution of this simulated ANM across a population of 10^4^ simulated women to empirical data on the ANM (*38*, *39*). On the basis of this, the ANM can be described as the threshold for stochastic growing follicle supply that can support any menstrual cycles, which ultimately depend on the availability and survival of at least one follicle through productive ovulation.

### Ovarian aging in terms of stochastic PFGA

While performing the study, consideration of how simulation data matched natural ovarian aging parameters led to the identification of a straightforward general relationship between the remaining ovarian reserve size (denoted *R*) and the onset of gaps in growing follicle supply. When the PF reserve size is *R*, the average time between PFGA events is approximately 1/(λ*R*), where λ ≈ 0.17/year. Converting this to a per day rate yields the simple rule that the average gap time between PFGA events is 2000/*R* days. Equivalently, the average number of PFGA events per day is approximately *R*/2000. We used this simple “rule of 2000” to generate a summary table (Table 1) of how numbers of remaining PFs and their loss patterns relate to the timing of the different ovarian aging stages: premenopause, the MT, and postmenopause.

**Table 1.** Summary of how follicle loss patterns relate to the premenopause, MT onset, and postmenopause states. Reserve size refers to size of the primordial follicle reserve; average gap refers to the average number of days where no primordial follicles begin to grow; possible gaps refer to the number of days where it is possible for zero primordial follicles to grow given available primordial follicles; and cycles refer to menstrual cycle patterns.

State	Reserve size	Average gap	Possible gaps	Cycles
Pre	>2000	<1 day	<7 days	Regular
MT	1000 to 2000	1 to 2 days	7 to 12 days	Irregular
Post	<1000	>2 days	>12 days	None

## DISCUSSION

Results presented here align with the proposed physiological sources of stochasticity [e.g., the integrated stress response pathway; (*15*, *28*)] and further validate the PF random walk model’s ability to precisely recapitulate ovarian aging at individual and population levels. The results also align well with historical studies that noted that stochastic models of PFGA agree with histological data on the decay of the ovarian reserve with age in both humans (*16*) and mice (*36*, *37*). Both Hirshfield (*27*) and Finch and Kirkwood (*26*) pointed out that the decline in the PF reserve over time is highly reminiscent of stochastic radioactive decay. Stochastic control of PFGA allows the robust supply of growing follicles for decades in the absence of a need for complex regulatory mechanisms to drive the process (*32*). Results provided here reinforce the concept that ovarian aging landmark stages instead occur emergently as a result of stochastic follicle behavior.

Key well-characterized features of reproductive aging in women are consistent with our model and its dependence on stochastic growing follicle supply. First, menstrual cycle length has been shown to exhibit an asymmetrical distribution (*40*) that is known to become more variable as women approach their forties. The asymmetrical “long right tail” of the menstrual cycle length distribution includes a fraction of cycles longer than 40 days across women. The authors of the original study posited that a “waiting time” can occur between “the end of one ovarian cycle and the initiation of the next ovarian cycle.” Stochastic growing follicle supply may contribute to rare long cycles and thus a waiting time within individuals. Harlow *et al.* (*41*) showed later that within-woman menstrual cycle length variance begins to rise late in the fourth decade, and this pattern was also detected by Guo *et al.* (*42*) using data from a separate cohort of women. These studies detected an increasing probability of both long and short cycles. Increasingly variable menstrual cycle lengths correspond to an increasingly variable (and increasingly interrupted) supply of growing follicles in this time frame. Our model output suggests that the onset of variable cycle length generally is likely to be influenced by the distribution of PF starting supply at birth as seen in the specific recapitulation of the timing of the MT where menstrual cycle length varies by 7 days.

In addition, while the supply of growing follicles has decelerated greatly by the times of the MT and menopause, PFGA still occurs within the model. Growing follicles are well-known to be present in the postmenopausal ovary (*43*), and there are reports where ovulation is confirmed to have occurred postmenopausally (*44*). While it is challenging to estimate the rate that growing follicles will survive to ovulate in the years approaching and after menopause, stochastic growing follicle supply provides an explanation for the well-known but “unpredictable” follicular activity even after menopause.

Deriving the human ANM distribution using a threshold “gap length” of 12 days where no PFs begin to grow extends prior work (*31*, *33*) that used a fixed threshold of 1000 remaining PFs. Unchanged model conditions also give rise to the reported natural distribution of MT onset when a shorter (and therefore earlier) gap length threshold of 7 days is set for growing follicle supply. These findings support the determination that the ovary is the organ of origin for the MT. Chronological aging is also likely to affect hypothalamus and/or pituitary function, and compromised function of these other regulators of the menstrual cycle may contribute to cycle changes that occur over time. However, the direct correspondence of stochastic growing follicle availability to the time-of-onset distribution for the MT suggests that as, with menopause (*45*), the major driver is indeed follicle loss within the ovary. The MT onset time distribution is shown to arise when the stochastic supply pattern of growing follicles reaches a point when too few growing follicles are available consistently enough to drive a regular menstrual cycle pattern. The ANM distribution is shown to correspond to the time that too few growing follicles are available consistently enough to support any (e.g., all but very rare) menstrual cycles.

In summary, stochastic growth activation behavior of primordial follicle units can be seen to be generative of the timing of the late physiological landmarks of reproductive aging within women and across the population of women. Despite its precisely matching retrospective datasets of the timing of reproductive aging landmarks, the model is in need of prospective validation. This will require improvements in live, noninvasive imaging of the ovary and also the ability to monitor the behavior of many immature follicles over lengthy time periods. A combination of imaging approaches, continued work elucidating biological pathways that control follicle loss, and continued mathematical analysis can move us closer to a comprehensive understanding of ovarian function and female reproductive aging.

## MATERIALS AND METHODS

Here, we describe our mathematical model of PF decay, MT onset, ANM, and variability across a population of women. These models build on our recent experimental, mathematical, and bioinformatics work (*15*, *28*, *31*, *32*, *35*). We also describe our method of stochastic simulation used to generate plots in this manuscript. MATLAB code and datasets from primary literature sources (*4*, *33*, *39*) have been deposited in a publicly available repository (figshare, https://doi.org/10.6084/m9.figshare.24454732).

### Mathematical model of PF decay

For a given woman, let *N* denote the number of PFs in her ovarian reserve at birth, which we call her starting supply [following (*33*), the ovarian reserve refers to the PFs in one ovary]. Suppose we label these PFs from 1 to *N* and let τ*_n_* denote the woman’s age when the PF labeled by *n* ∈ {1, …, *N*} leaves the reserve (either though growth activation or atresia). We call τ_1_, τ_2_, …, τ*_N_* the PFGA times. Following (*16*, *31*, *36*, *37*), we assume

$$\tau_{1},\tau_{2},\ldots ,\tau_{N}\;\text{are independent and identically distributed}$$ (1)

Let *S*(*t*) denote the survival probability (i.e., the complementary cumulative distribution function) of each PFGA time, which means

$$S\left(t\right)=\mathbb{P}\left(\tau_{n}>t\right),\;\;t\geq 0$$ (2)

In words, *S*(*t*) is the probability that a given PF leaves the ovarian reserve after age *t* ≥ 0.

We assume that *S*(*t*) is given by the random walk model in (*31*), which means

$$S\left(t\right)=\sum_{k=1}^{\infty }A_{k}e^{-\lambda_{k}t}$$ (3)

where

$$\lambda_{k}≔\frac{Dk^{2}\pi^{2}}{4}+\frac{V^{2}}{4D}$$

$$A_{k}≔\frac{2\pi k[1-{\left(-1\right)}^{k}e^{\frac{-V}{D}}]}{\pi^{2}k^{2}+V^{2}/D^{2}}e^{\frac{V}{2D}}\text{sin}\left(\frac{k\pi }{2}\right),\;\;k\geq 1$$

where the parameters *D* = 0.004 year^−1^ and *V* = 0.051 year^−1^ were chosen to fit the PF decay data reported in (*33*). The infinite series in Eq. 3 is dominated by the first term except at early ages. In particular, we have that *S*(*t*) in Eq. 3 decays exponentially as the age *t* grows, so that it is well approximated by

$$S\left(t\right)\approx A_{1}e^{-\lambda_{1}t}\;\;\text{if}\;t\geq 25\;\text{years}$$

where λ_1_ = 0.17 year^−1^ and *A*_1_ = 21.4.

### Modeling MT onset and ANM

STRAW+10 criteria (*22*) were used to produce the available time distribution for MT onset (*4*). These were “a persistent difference of at least 7 days in the length of consecutive menstrual cycles with persistence defined as recurrence within 10 cycles of the first variable length cycle.”

For a given woman in our model, we define the onset of the MT (denoted *T*_MT_) as the first time she goes a critical time *t*_1_ = 7 days without PFGA

$$T_{\text{MT}}=\text{inf}\left\{t>t_{1}:R\left(t\right)=R\left(t-t_{1}\right)\right\}$$ (4)

where *R*(*t*) is the size of the reserve at age *t* ≥ 0

$$R\left(t\right)=\sum_{n=1}^{N}1_{\tau_{n}>t}$$ (5)

where 1_τ*_n_*>*t*_ denotes the indicator function on the event τ*_n_* > *t* (i.e., 1_τ*_n_*>*t*_ = 1 if τ*_n_* > *t* and 1_τ*_n_*>*t*_ = 0 otherwise).

Similarly, for a given woman, we define the onset the menopause age (denoted *T*_ANM_) as the first time she goes a critical time *t*_2_ = 12 days without PFGA

$$T_{\text{ANM}}=\text{inf}\left\{t>t_{2}:R\left(t\right)=R\left(t-t_{2}\right)\right\}$$ (6)

### Modeling starting supply variability

The mathematical modeling described above concerns a single woman with a given starting supply (ovarian reserve size at birth) *N*. Starting supplies are known to vary widely between women (*33*, *34*). It was shown in (*31*) that the starting supply population data reported in (*33*) is well described by the following log-normal probability distribution

N=exp(μ+σZ) (7)

where *Z* is a standard normal random variable, and μ = 12.686 and σ = 0.497.

Notably, compelling evidence is available that supports a positive relationship between an individual’s starting supply and the expected duration of ovarian function [e.g., the expected timing of onset of the MT and the ANM for that individual (*8*, *9*)].

### Stochastic simulations of MT onset and ANM

The simulated population distributions of MT onset and menopause age shown in Results are simulated by the following method. For each simulated woman, her starting supply *N* is sampled according to the log-normal distribution in Eq. 7. Then, her PFGA times τ_1_, …, τ*_N_* are simulated according to Eqs. 1 to 3. These PFGA times then yield the reserve size time course *R*(*t*) according to Eq. 5, which then yields the MT onset age *T*_MT_ and the menopause age *T*_ANM_ according to Eqs. 4 and 6. Repeating this procedure for 10^4^ independent simulated women yields the simulated population distributions shown in Results. The plots of stochastic PFGA gaps for a single woman in Results are obtained similarly, where we define the sequence of gaps *g*_1_, …, *g_N_* occurring at ages τ_1_, …, τ*_N_* according to *g*_1_ = 0 and

$$g_{n}=\tau_{n}-\tau_{n-1},\;\;n=2,\ldots ,N$$
